# Usefulness of Neurosonogram in Critical Ill Neonates

**DOI:** 10.7759/cureus.24882

**Published:** 2022-05-10

**Authors:** A Rupesh Rao, Amar Taksande

**Affiliations:** 1 Pediatrics, Jawaharlal Nehru Medical College, Wardha, IND

**Keywords:** high risk newborn, pediatric ultrasound, high risk neonates, critical ill neonates, neurosonogram

## Abstract

The Cranial Neurosonogram is the preferred method for viewing the infant's brain. Ultrasound tools are portable and may be used at the NICU bedside. This corresponds to the concept of point-of-care testing. The difficulties associated with moving newborns to CT or MRI rooms are eliminated. Furthermore, ultrasound is less expensive than CT, has no radiation impact, and does not require sedation, which is required for MRI. Cranial sutures are still open in newborns, allowing us to glimpse within the brain using ultrasonography. A radiologist or neonatologist specializing in that profession should do the neurosonogram. The majority of the time, the course of therapy and subsequent care of the patient can be based on a Neurosonogram finding. Regardless of weight, height, or gestational age, any neonate who has a higher risk of morbidity or death due to fetal, placental, or maternal factors is classified as critically unwell. A sick neonate is defined as any neonate, regardless of birth weight, size, or gestational age, who has a greater than average risk of morbidity or mortality due to fetal, maternal, or placental anomalies or an otherwise compromised pregnancy within the first 28 days of life.

## Introduction and background

The Cranial Neurosonogram is the preferred method for viewing the infant's brain. The advantages of a cranial neurosonogram are numerous: it can be performed at the bedside, is reasonably safe, and may be performed whenever necessary; it aids in the detection of continuing brain growth and the development of lesions [1,2]. In the newborn intensive care unit (NICU), ultrasound is routinely utilized for cranial imaging, and Ultrasound tools are portable and may be used at the NICU bedside. Furthermore, ultrasound is less expensive than computerized tomography (CT), has no radiation impact, and does not require sedation, which is required for magnetic resonance imaging (MRI). Many sutures are still open in newborns, allowing us to glimpse within the brain using ultrasonography. A radiologist or neonatologist specializing in that profession should do the neurosonogram. The majority of the time, the course of therapy and subsequent care of the patient can be based on a neurosonogram finding [3].

Neursonogram helps in the early assessment and prognosis of the neurological condition of these critically ill neonates. The latest advanced ultrasound technology helps diagnose various diseases based on cranial images. Neurosonogram detects changes like calcification, ischemia, cystic lesion, and hemorrhagic lesions inside the brain [4]. In many NICU settings, they are doing an early screening of neonates, mainly the preterm, for any neurological abnormality. When performed according to protocol, neurosonography is a reliable technique for most newborn illnesses. With the help of the ultrasound, which is portable and can be done at the bedside, a neurosonogram is widely used as an initial screening tool of the neonatal brain and can be repeated as many times as possible.

By increasing the fair and efficient use of neurosonogram in the neonates to screen the cranial abnormalities, various conditions causing severe morbidity or mortality can be detected early in their course and thereby be efficiently managed for better prognosis.

## Review

History

Significant technological advances had occurred since the late 1950s when Leksell first used ultrasound to assess intracranial contents [5]. Using A mode Echo Encephalography, the brain was the first organ systematically studied by ultrasound. Encephalography as a model was developed in the 1950s to identify midline structure and changes and provide a rough estimate of ventricular size. In the 1960s, 2 D echo encephalography was introduced, allowing for improved observation of ventricular size and intracranial spatial connection. Scanning in amplitude mode (A-mode) and static Grayscale imaging are of historical importance.

Detailed cross-sectional images of the infant's brain were first published by Kossoff in 1974, using an automated patch scanner, the Octoson. Sector and linear, high frequency, compact head transducers that easily fit over the fontanelle have now replaced linear array systems with big transducers.

The ultrasound probe comes in different frequencies, more frequency probes have better resolution and good quality image, but less penetration opposite is true for low frequency. As a result, more frequency transducers are employed in newborn brain exams. Since Leksell's use of ultrasound in 1956 (A-mode) and the pioneering work of Kossoff and colleagues [5] and Garrett and colleagues [6] on ultrasound of the normal and hydrocephalic fetal brain, image resolution with real-time Grayscale imaging has substantially enhanced [7].

The first reports of ultrasound detection of IVH appeared in the late 1970s (Heinburger, 1978, Johnson, 1979, Pape, 1979), and modifications and improvements to this method rapidly occurred.

Initially, the anterior and temporal fontanelles of the skull were used as windows; however, Dewbury and Aluwikaee, and Babcock reported for the first time in 1980, using the mastoid fontanel as the bone-free window. Transfontanel ultrasonography has become a common imaging tool for the newborn and infantile brain and is frequently employed. Color and pulsed wave Doppler have been added to ultrasound imaging through the skull, increasing its versatility [5].

Newer advances in image recording technology have added a new dimension to newborn brain ultrasound examination, transitioning from static classic picture film and videotape to the latest digital file on a hard disc with the ability to post-process the images. The major neuropathologic patterns of hypoxic-ischemic cerebral injury are shown in Table 1 [8,9]. Risk factors for critically ill neonates is mentioned din table 2.

**Table 1:** Major neuropathologic changes

Pattern of injury	Gestational age		Anatomic distribution
	Preterm neonates	Full-term neonates	
Selective neuronal necrosis	Present	Present	Cortex, brain stem, hippocampus
Parasagittal	Absent	Present	Parasagittal cortex
Focal or multifocal necrosis	Present	Present	Cerebral cortex
Periventricular Leukomalacia	Present	Absent	Periventricular white matter

**Table 2:** Risk factors for critically ill neonates

Maternal factors
Age at delivery
Poverty
Smoking
Drug/alcohol use
Fetal conditions
Multiple gestations
Macrosomia
Abnormality of fetal heart rate or rhythm: hydrops, asphyxia, congestive heart failure, heart block
Polyhydramnios
Oligohydramnios
Perinatal factor

Congenital Abnormalities

A congenital abnormality is any change in normal anatomic structure at birth. It might be substantial or little, independent or part of a wider constellation of flaws, with an obvious or unclear source. Several genetic and environmental etiologies are clearly defined, but the underlying cause of roughly half of all birth abnormalities is unknown. Four types of factors cause brain malformations: (a) chromosomal abnormalities, (b) single gene defects, (c) extrinsic teratogens, and (d) unknown. The latter is the most common, accounting for 75% of all malformations [10].

During pregnancy, the dilated cerebral ventricles (ventriculomegaly > 10mm) increase the risk of hydrocephalus, dandy-walker cyst, and agenesis of the corpus callosum. If isolated, transient ventriculomegaly is normally benign and common; however, chronic and recurrent ventriculomegaly necessitates the evaluation of cranial and extracranial defects. Neurosonogram is performed serially after birth to assess growth and course [11].

Hydrocephalus

The most common neurosurgical consultation for newborn patients is to evaluate and treat enlargement of the ventricular system. The clinical signs of progressive enlargement of the ventricular system include an excessive increase in the head circumference (HC), fullness in the anterior fontanelle (especially when the patient is upright, the venous and fontanelle pressures should be low), episodic apnea and bradycardia, general lethargy, and abnormalities of ocular movement, especially restricted upgazed [12].

**Figure 1:**
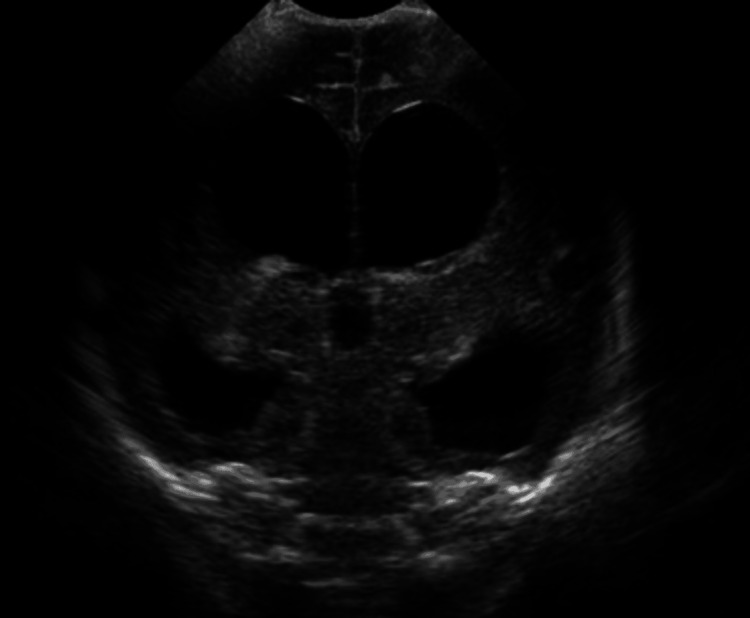
Neurosonogram shows dilatation of ventricular system Credit: Image taken by authors at the hospital.

The most crucial difference between progressive and static problems is ventricular volume. Using repeated neurosonogram exams, this is quite simple to identify. Figure 1 shows dilatation of ventricular system in meurosonogram. The recent addition of resistive index (RI) measurements adds a physiologic dimension to neurosonogram anatomic images. One of the problems with assessing the growth of ventricular size in the neonate is that the enlargement of the ventricles may at least partially reflect the loss of tissue in the brain rather than an increase in pressure. Nevertheless, assessing for macrocephaly is complicated by the changing growth rate of the normal neonatal head. An HC growth of less than 1 cm/week is acceptable, and an HC growth of more than 1.5 cm/week is considered excessive. Fluid in the subarachnoid space outside the brain, sometimes termed "Benign External Hydrocephalus," is probably a normal variant. The increased extra-axial fluid collection is generally in the frontal regions [13].

Children with non-hemorrhagic hydrocephalus, like aqueduct stenosis, posterior fossa cysts, holoprosencephaly, or hydranencephaly, must be operated on within 1 to 2 days following birth. Nearly all acquired hydrocephalus in the premature infant is post-hemorrhagic. The risk of PHH correlates with the severity of intraventricular hemorrhage. For the evaluation of a case, a neurosurgeon consultation is mandatory. An examination often includes a neurosonogram of the skull to determine the size of the ventricles and a lumbar puncture to detect CSF pressure. The RI of the Anterior cerebral artery may be measured during a neurosonogram (RI is defined as systolic - diastolic blood velocity/systolic blood flow velocity). Concern regarding hemodynamic compromise is raised either by an RI above the normal value of 0.5 to 0.7 or a rise in RI with gentle compression of the anterior fontanelle [12].

Aqueduct Stenosis

It is a common congenital cause of hydrocephalus that is distinguished by significant dilation of the third and lateral ventricles and the lack of all other anatomical abnormalities. Aqueduct stenosis can mimic holoprosencephaly when the lateral ventricles are severely dilated, and the cerebral mantle is small. Holoprosencephaly comprises fused thalami and a missing third ventricle, whereas aqueduct stenosis has splayed thalami and an enlarged third ventricle. Mastoid fontanelle imaging gives an excellent picture of brainstem anatomy and can assist in distinguishing between these two abnormalities, which have different prognoses and treatments [12].

Dandy-Walker Malformation (DWM)

DWM is often defined as partial or total vermian agenesis with hypoplastic cerebellar hemispheres, fourth ventricle cystic expansion, and posterior fossa expansion with raised tentorium and transverse sinus insertion. Cerebellar hemispheres are hypoplastic as well, and they border the petrous ridges. The posterior fossa is occupied by a large cyst resembling an enlarged fourth ventricle, whereas the cisterna magna is effaced. In nearly 80% of untreated cases, hydrocephalus develops. Diagnosis of DWM is critical for therapy and prognosis. Once again, mastoid fontanelle scanning of the posterior fossa better represents the cerebellum and perception of tiny clefts that are not easily apparent from the anterior fontanelle [13].

Birth Injury

The most prevalent type of head damage in a newborn is cephalhematoma. It affects up to 2.5 percent of live newborns and is most frequent following instrumental delivery, particularly vacuum extraction. Neurosonogram will detect co-existing cerebral fluid and the boundaries of a suspected cephalhematoma [14].

Intracranial Fluid Collections

Subdural hemorrhage is the most common intracranial abnormality following birth trauma. Malpresentation, prolonged labor, and forceps delivery commonly predispose to it. Subarachnoid hemorrhages are uncommonly associated with birth trauma in term neonates, and subarachnoid adhesions may develop later, leading to hydrocephalus. Extradural hemorrhage is rare and associated with birth trauma and may present with a skull fracture [15].

Vascular Anomalies of the Neonatal Brain

Anatomical, pathophysiological, and clinical abnormalities such as cerebral arteriovenous shunts, Dural meningeal arteriovenous shunts, and artery aneurysms are vascular malformations. Many malformations detected in neonates are connected with developmental abnormalities due to the brain's specific vulnerability during the perinatal period. Before birth, two types of defects are discovered: aneurysmal abnormalities of the vein of Galen and defective Dural sinuses [16].

Aneurysmal Malformations of the Vein of Galen

A neurosonogram or MRI can reveal a vein of Galen abnormality as soon as the 28th week of gestation. They appear as a spherical cerebral mass behind the third ventricle with flow resembling an arteriovenous shunt. Possible symptoms include heart failure with tachycardia, ventricular extrasystoles, and tricuspid regurgitation. Vein of Galen abnormalities is extremely uncommon congenital connections of intracranial vessels. They arise during the first months of development and are associated with chronic venous anomalies such as a missing straight sinus and persisting falcine and occipital sinuses. Vein of Galen Aneurysmal causes venous hemodynamic abnormalities that result in macro crania or ventriculomegaly. The ventricular enlargement is caused by venous sinus obstruction rather than aqueduct compression; later, it can be secondary to brain atrophy [12].

Periventricular Leukomalacia (PVL)

It is a lesion observed mostly in premature newborns. It is most likely the neuropathologic lesion responsible for many neurological, motor, and sensory deficits and inadequacies seen in preterm births. The abnormalities were found in the periventricular white matter dorsolateral to the lateral ventricles, namely anterior to the frontal horns (near the foramen of Monro) and lateral to the occipital horns [17].

Many studies have shown that cystic PVL can be detected using a neurosonogram since 1983. When attempting to diagnose PVL, serial ultrasound scans over an extended period (several weeks) are needed. After a known provocation, a region of echo density occurs within 24 and 48 hours, but cysts do not grow for another 2-4 weeks. Although there is no universally agreed classification scheme for PVL, the De Vries classification is widely used. The conventional categorization criteria for PVL by ultrasonographic imaging is the development of echogenicity in the periventricular white matter during the first few weeks after birth, both with and without cysts. Ventriculomegaly due to periventricular white matter atrophy is common within a few weeks [18].

Periventricular Haemorrhagic Infarction (PVHI) is an acquired brain lesion that significantly affects the neurodevelopmental result in prematurely born babies. PVHI has traditionally been regarded as the most extreme (grade IV) type of germinal matrix-intraventricular hemorrhage (GM-IVH). However, research has revealed that PVHI is a consequence of GM-IVH, which comes from a hemorrhagic transition of a venous infarct rather than a normal parenchymal increase of the intraventricular fluid. For several years, the neonatal neurosonogram (NUSG) has been the primary method for diagnosing GM-IVH and PVHI in premature babies. Haim Bassan, Carol B. Benson et al. demonstrated that periventricular hemorrhagic infarction could be scored using a sonographic scoring method [19]. Their study attempted to identify the size and topography of PVHI in the modern ages and outline the normal neurosonogram findings in PVHI progression to develop a neurosonogram -based scoring system expressing the structural intensity of PVHI during the early neonatal period.

Intracranial Hemorrhage

**Figure 2:**
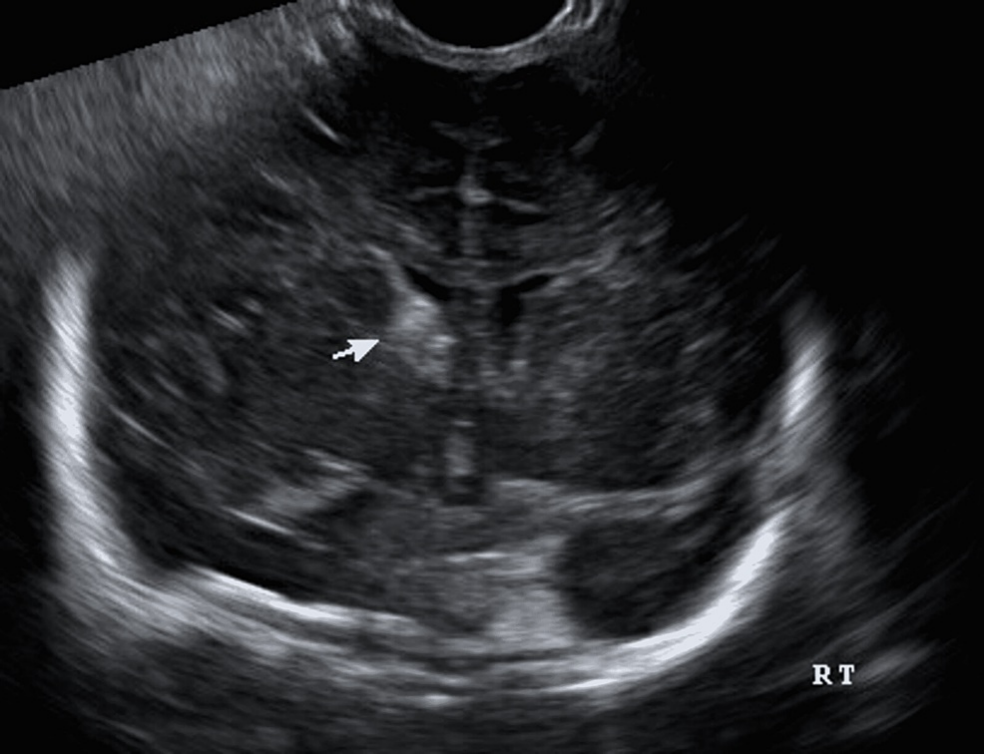
Neurosonogram shows Germinal matrix hemorrhage. Credit: Image taken by authors at the hospital.

Germino-matrix-haemorrhage (GMH): GMH is still most prevalent in preterm babies, with 15% to 20% in babies delivered at 32 weeks gestation, while it is rare in term births. These two sorts of newborns have different aetiologies and pathophysiology. GMH/IVH in the preterm baby is caused by the weak involuting vessels of the subependymal germinal matrix, which is found in the caudothalamic groove. Intravascular factors play an important role in the pathogenesis of GMH/IVH, mainly the modulation of cerebral perfusion (e.g., cerebral blood flow and pressure) within the fine vasculature of the germinal matrix platelet-capillary interactions and coagulation disruptions. The fragility of vessels in the germinal matrix and their susceptibility to hypoxic-ischemic insult are vascular pathogenetic factors. Extravascular causes include supportive tissue features, prolonged fibrinolytic activity, and a potential reduction in tissue pressure post-natally. Figure 2 shows Germinal matrix hemorrhage on neurosonogram.

Primary IVH in the term neonate generally starts in the choroid plexus or in conjunction with venous (sinus) thrombosis and thalamic infarction. However, it can also start in a small remnant of the subependymal germinal matrix. Injury (such as a difficult birth) or prenatal hypoxia is the most common cause of IVH in a term newborn. GMH/IVH has two main complications: periventricular hemorrhagic infarction (PVHI) and post-hemorrhagic ventricular dilation (PVD). Because of its portability, high resolution, and absence of ionizing radiation, the neurosonogram is considered the neuroimaging modality of choice for diagnosing GMH/IVH. CT and MRI continue to be better for identifying various types of cerebral bleeding, such as primary subarachnoid, convexity, posterior fossa subdural or epidural hematomas, and distinguishing between hemorrhagic and ischemic parenchymal infarction [20].

Subdural Hemorrhage

Obstetric technique advancements and a reduction in mechanical birth injuries have significantly decreased the rate of major subdural hemorrhage. Subdural bleeding can occur in preterm and term neonates and is produced by a laceration of the major veins and sinuses, which is frequently associated with a rupture of the dura or Dural reflections (e.g., falx, tentorium) covering the cerebral hemispheres or cerebellum. Excessive head shaping can contribute to the development of a subdural hematoma. Using the conventional neurosonogram technique via the anterior fontanelle, diagnosing posterior fossa hemorrhage and minor lesions can be challenging across the cerebral convexities. Imaging the posterior fossa via the posterolateral fontanelle can enhance visualization. CT is better at detecting supratentorial lesions, whereas MRI is better at detecting posterior fossa hemorrhage.

Primary Subarachnoid Hemorrhage (SAH)

SAH is usually detected in the subarachnoid area above the cerebral convexities and in the posterior fossa, where bleeding inside the subarachnoid space is not caused by subdural hemorrhage, IVH, or cerebellar hemorrhage. Subarachnoid hemorrhage in neonates is usually self-limiting and venous, originating from tiny vessels in the leptomeningeal plexus or bridging veins inside the subarachnoid space. Clinically, it might be asymptomatic or appear as seizures, and in rare cases, as abrupt neurologic impairment. The diagnosis is predicated on the observation of consistently blood-stained CSF following a lumbar puncture in a baby after CT had ruled out all other sources of cerebral bleeding. Primary subarachnoid hemorrhage CT scans frequently reveal blood in the superior longitudinal fissure and sulci.

Intracerebellar Hemorrhage

Primary bleeding into the cerebellum is a relatively frequent lesion, particularly in preterm newborns. The comprehensive use of neurosonogram through the posterolateral fontanelle in preterm neonates has revealed that all these conditions were formerly undiagnosed in living newborns. Possible factors include mechanical laceration of the cerebellum or sinuses, venous infarction, IVH enlargement, or severe subarachnoid hemorrhage into the cerebellum. Clinically, there is typically a catastrophic hemodynamic degradation. There is typically a history of difficult breech birth in term babies, followed by the emergence of neurologic symptoms associated with brainstem compression. The lesion could be assumed based on the experience, and physical characteristics mentioned previously. Traditional neurosonogram across the anterior fontanelle has little utility for detecting intracerebellar hemorrhage, but acoustic windows may be useful.

## Conclusions

Neurosonogram is an excellent technique for screening the neonatal brain in its early stages. Despite the ubiquitous availability of ultrasound machines in institutions, neurosonogram penetration remains low in neonatal intensive care units. Neurosonogram (NUSG) provides access to bedside imaging of the neonatal brain. It is a reliable tool for detecting congenital and acquired abnormalities of the neonatal brain and the most common brain injury patterns in preterm and term neonates. It detects most hemorrhagic, ischemic, cystic brain lesions, calcifications, cerebral infections, and major structural abnormalities in preterm and term neonates.
